# Evaluation of agmatine efficacy in contrast-induced nephropathy: experimental evidence in rat model

**DOI:** 10.1590/acb412426

**Published:** 2026-05-18

**Authors:** Murat Yeniçeri, Süleyman Baş, Mustafa Can Şenoymak, Musa Salmanoğlu, Alpaslan Tanoğlu

**Affiliations:** 1 University of Health Sciences – Kartal Dr. Lutfi, Kirdar City Hospital – Department of Rheumatology – Istanbul – Turkey.; 2 University of Health Sciences – Department of Internal Medicine – Sancaktepe Sehit Prof. Dr. Ilhan Varank Training and Research Hospital – Istanbul – Turkey.; 3 University of Health Sciences Department of Internal Medicine – Sultan 2 – Abdulhamid Han Training and Research Hospital – Istanbul – Turkey.; 4 Bahçeşehir University – Göztepe Medical Park Hospital – Department of Internal Medicine – Division of Gastroenterology – Istanbul – Turkey.

**Keywords:** Agmatine, Iohexol, Contrast Media

## Abstract

**Purpose:**

To investigate the biochemical and histopathological efficacy of agmatine as a protective agent in experimental rat contrast-induced nephropathy (CIN) model.

**Methods:**

Twenty-eight male Sprague Dawley rats were randomized into four groups: sham (no intervention), contrast agent (single dose 10 mL/kg iohexol iv), contrast agent + agmatine (single dose 10 mL/kg iohexol iv + 20 mg/kg po agmatine three days) and contrast agent + placebo (single dose 10 mL/kg iohexol iv + 1 mL of 0.9% saline ip). Biochemical and histopathological evaluations were accomplished on the fourth day, including measurements of cystatin-C, malondialdehyde (MDA), glutathione peroxidase (GPx), interleukin (IL)-1 β, superoxide dismutase (SOD), and tumor necrosis factor (TNF)-α.

**Results:**

The contrast agent group exhibited elevated cystatin-C, IL-1β, TNF-α, and MDA levels, with decreased SOD and GPx levels. The agmatine treatment group showed decrease in cystatin-C, IL-1β, TNF-α, and MDA, along with increased GPx and SOD levels. Histopathological evaluation revealed nephrotoxic effects in the contrast agent and placebo groups, whereas agmatine-treated rats displayed a significant reduction in tubular damage.

**Conclusion:**

Agmatine administration along side contrast exposure demonstrated protective effects against CIN, evidenced by biochemical and histopathological improvements.

## Introduction

In recent years, the frequent utilization of radiological interventional procedures and imaging methods gives rise to contrast induced nephropathy (CIN) as a significant complication. CIN is defined as the acute renal injury that occurs following exposure to intravenous contrast material, after excluding other etiological factors that could lead to acute kidney injury^1^. The true incidence of CIN is unknown although various rates between 1.9 and 36.9% are provided in the studies^2^. The nephrotoxicity of iodinated contrast agents has been substantiated through numerous investigations^1,3,4^. According to these studies, iodinated contrast agents induce vasoconstriction, leading to the development of acute tubular necrosis. Other underlying factors include oxidative stress, apoptosis, and inflammation, possibly stemming from alterations in mediators such as adenosine, endothelin, or nitric oxide and direct toxic effects of the contrast agent^3-5^. From this perspective, antioxidant and anti-inflammatory therapy may hold a significant position considering the pathogenesis of CIN.

Agmatine is a decarboxylation product of arginine and represents a polyamine molecule present in various tissues of mammals. It has gained attention due to its reported vasodilatory, antioxidant, and anti-inflammatory effects^6,7^. These effects primarily stem from agmatine’s modulation of nitric oxide synthesis while it manifests varying effects through distinct mechanisms in multiple target tissues^6,8^. Agmatine serves as a dual regulator of nitric oxide, inhibiting inducible nitric oxide synthase to control excessive nitric oxide (NO) production and suppress inflammation while concurrently activating endothelial nitric oxide synthase to promote vasodilation. It also may contribute to vasodilation by activating imidazoline 1 and alpha 2 receptors^8^.

Several recent studies support its protective role in nephrotoxic and inflammatory conditions, including agmatine-related research providing strong evidence for renoprotective effects in several models. Sugiura et al.^8^ reported that agmatine improved renal function and histology in a rabbit CIN model, although species differences may influence results. Similarly, Hassan et al.^9^ demonstrated that agmatine significantly reduced oxidative stress and inflammation in methotrexate-induced nephrotoxicity. For instance, Sugiura et al8. demonstrated that agmatine at a similar dose exerted protective effects in ischemia/reperfusion–induced renal injury, while Hassan et al.9 and Salihoglu et al^10^. reported significant improvements in oxidative stress markers and renal function parameters in drug-induced nephrotoxicity models using comparable dosing regimens. Kamel et al.^11^ confirmed protective effects of agmatine in diabetic nephropathy, showing improvements in renal biomarkers and histology.

Moreover, agmatine treatment improved biochemical and histopathological outcomes in cerulein-induced acute pancreatitis^12^, further supporting its anti-inflammatory and antioxidant properties across different organ injury models. Previous studies have suggested that agmatine might hold promise as a protective agent against renal injury in various experimental models^13-16^.

This study aimed to investigate the biochemical and histopathological effects of agmatine administered as a protective agent in rat models with CIN.

## Methods

### Study design

The investigation was undertaken in the Experimental Medicine Research and Application Center, University of Health Sciences, Istanbul, Turkey. Ethical approval for the study was obtained from the University of Health Sciences Animal Experiments Local Ethics Committee (approvel date and number: 2020-03/13). The study adhered to the principle soutlined by the Council of Europe (European Convention for the Protection of Vertebrate Animals Used for Experimental and Other Scientific Purposes), pertaining to the safeguarding of vertebrate animal semployed for experimental and scientific objectives.

In this investigation, a cohort of 28 male Sprague Dawley rats with body weights ranging from 250 to 350 grams was employed. The rats were housed within cages, adhering to a controlled 12-hour light-dark cycle and maintained in a climate-controlled environment at 24 °C. Preceding the administration of injections, the rats were granted *ad libitum* access to both food and water. Food access was discontinued 12 hours prior to sacrification.

The rats were randomized into four groups, consisting of seven in each group:

- Group 1: sham group; no intervention was applied to the rats in this group;
- Group 2: contrast agent group. Iohexol contrast agent was injected intravenously via the tail vein at the dose of 10 mL/kg on the second day of the study, and the rats were sacrificed on the fourth day^16^;
- Group 3: contrast agent + agmatine group. On the second day of the study, rats were intravenously injected with iohexol contrast agent at the dose of 10 mL/kg via the tail vein. Agmatine was administered orally at the dose of 20 mg/kg on the first, second, and third day of the study, and the rats were sacrificed on the fourth day;
- Group 4: contrast agent + placebo group. On the second day of the study, iohexol contrast agent was injected intravenously via the tail vein at the dose of 10 mL/kg. On the first, second and third day of the study, 1 mL of 0.9% saline as placebo was administered intraperitoneally, and the rats were sacrificed on the fourth day.

### Biochemical and histopathological evaluation

On the fourth day, all rats underwent anesthesia through intraperitoneal injection of a xylazine (10 mg/kg) and ketamine (75 mg/kg) combination. A midline laparotomy was subsequently performed, and blood samples were promptly collected from the inferior vena cava. Following laparotomy, right kidney tissues were excised, and the rats were humanely sacrificed. The collected blood samples were subjected to centrifugation at 3,000 rpm for 10 minutes and subsequently stored at the temperature of -70 °C until further analysis. Subsequently, concurrent measurements of cystatin-C (Cys-C), malondialdehyde (MDA), glutathione peroxidase (GPx), superoxide dismutase (SOD), tumor necrosis factor alpha (TNF-α), and interleukin-1 beta (IL-1β) were conducted on all blood samples. The assays were performed using BT Lab rat solid phase enzyme-linked immuno-sorbent assay (ELISA) kits, following manufacturers’ instructions and guidelines.

For histopathological assessment, the right kidney was fixed in a 10% formaldehyde solution, followed by parafin embedding. Kidney samples were sectioned at 4 microns, stained with hematoxylin and eosin. Renal histopathological evaluation was performed using a semiquantitative scoring system based on the percentage of affected renal tissue. Tubular injury was assessed by examining tubular degeneration, necrosis, inflammatory cell infiltration, and cast formation in hematoxylin and eosin–stained sections. The severity of renal injury was graded as follows:

- Score -: no histopathological damage;
- Score +: mild damage affecting < 25% of the renal tissue;
- Score ++: moderate damage affecting 25–50% of the tissue;
- Score +++: severe damage affecting > 50% of the tissue.

All histopathological evaluations were conducted by an experienced pathologist blinded to the experimental groups to minimize observer bias.

### Statistical analyses

In data analysis, Statistical Package for the Social Sciences version 25 software was employed. Descriptive analyses (frequency distributions, percentages, means, standard deviations), normality distribution assessed via Kolmogorov-Smirnov’s test, and graphical representations were utilized as statistical methods for result analysis. In cases in which the results exhibited a normal distribution, analysis of variance (post hoc Bonferroni’s test) was employed; alternatively, in cases in which normality was not observed, the Kruskal-Wallis’ and Mann-Whitney’s U test were applied. The outcomes were evaluated at a significance level of *p* < 0.05, within a 95% confidence interval.

## Results

The levels of Cys-C, GPx, IL-1β, SOD, TNF-α, and MDA obtained from the serum samples of the rats are presented in Table 1.

**Table 1:** Biochemical parameters of study groups.

Parameters	Group 1 sham	Group 2 C.A.	Group 3 C.A. + agmatine	Group 4 placebo	*P* -value
Cys-C (ng/mL)	5.30 ± 0.39*	7.40 ± 0.78*	5.21 ± 0.31*	7.03 ± 0.83*	< 0.001^a^
GPx (ng/mL)	18.79 ± 0.97*	15.39 ± 0.69*	18.05 ± 0.83*	15.66 ± 1.02*	< 0.001^a^
IL-1β (pg/mL)	504.44 ± 41.24*	639.29 ± 31.74*	556.62 ± 20.12*	615.73 ± 45.08*	< 0.001^a^
SOD (ng/L)	1.13 ± 0.12*	0.76 ± 0.04*	0.89 ± 0.05*	0.84 ± 0.06*	< 0.001^a^
TNF-α (ng/mL)	48.71 ± 8.24*	67.76 ± 3.65*	51.04 ± 4.51*	63.56 ± 3.70*	< 0.001^a^
MDA (nmol/mL)	0.79 ± 0.05*	1.29 ± 0.07*	1.19 ± 0.08*	1.27 ± 0.07*	< 0.001^a^

Cys-C: cystatin-C; GPx: glutathione peroxidase; IL: interleukin; SOD: superoxide dismutase; TNF: tumor necrosis factor; MDA: malondialdehyde; C.A.: contrast agent. Source: Elaborated by the authors.

**Figure 1:**
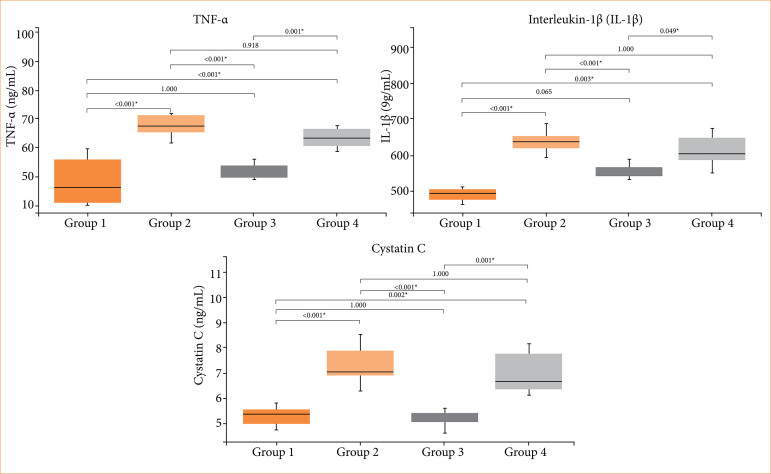
Comparison of tumor necrosis fator (TNF)-α, interleukin (IL)-1β, and cystatin-C (cyS-C).

Source: Elaborated by the authors.

**Figure 2:**
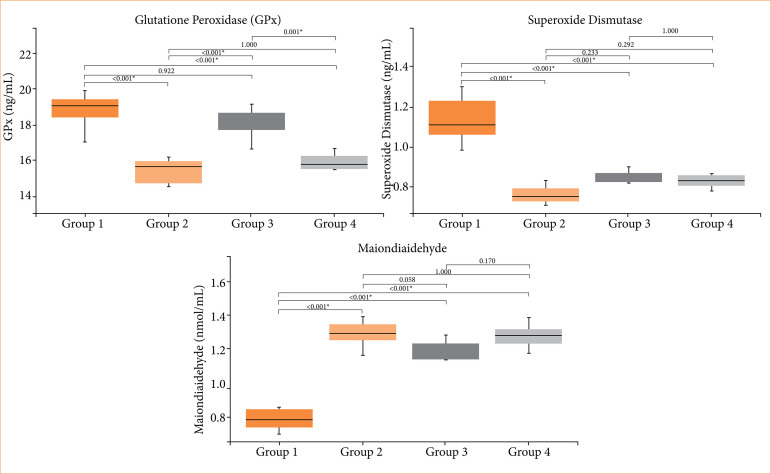
Comparison of glutathione peroxidase (GPx), superoxide dismutase (SOD), and malondialdehyde (MDA).

Source: Elaborated by the authors.

The administration of contrast agent resulted in a notable elevation in cys-C, IL-1β, TNF-α, and MDA levels when compared to the sham group (group 1) of rat subjects. Furthermore, there were significant decreases observed in SOD and GPx levels in the group exposed to the contrast agent (group 2). Statistical analysis did not reveal any significant difference between the placebo (group 4) and the contrast agent group (group 2). In the cohort subjected to the administration of both the contrast agent and agmatine (group 3), substantial reductions were evident in the levels of cys-C, IL-1β, and TNF-α in comparison to the contrast agent group (group 2) and placebo (Fig. 1). Conversely, although a reduction was expected in group 3, MDA levels remained higher than in the sham group, and no statistically significant differences were observed among groups 2, 3 and 4 (Fig. 2). SOD and GPx levels were significantly decreased in groups 2 and 4 compared to the sham group, consistent with the induction of oxidative stress. In group 3, an improvement in GPx activity was observed compared to the C.A. and placebo groups, but no improvement was noted in SOD activity, and the differences among groups 2, 3 and 4 were not statistically significant. These results suggest that agmatine treatment may exert a partial protective effect on antioxidant capacity, although it does not fully restore oxidative balance.

In the histopathological evaluation, kidney tissues of the sham group exhibited normal histological structure. Contrast agent administration caused pathological changes indicative for nephrotoxicity. Severe vacuolization, tubular dilatation and casts were observed in tubules.

**Figure 3:**
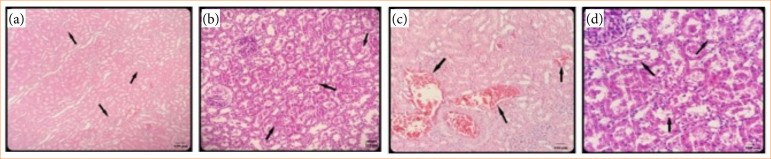
Light micrograps of sections from rat kidneys.

Source: Elaborated by the authors.

Most of the renal corpuscles lost their normal morphology, and there was severe glomerual degeneration. The histopathological scores of the placebo group were similar to the contrast agent group (Table 2). In contrast, rats receiving concurrent agmatine treatment demonstrated a remarkable reduction in the extent of tubular damage with almost normal renal corpuscle morphology, as depicted in Fig. 3.

**Table 2:** Histopathological findings according to experimental groups.

Groups	Mononuclear cell infiltration	Tubular degeneration	Tubular necrosis	Casts in tubular lumen
1 (sham)	-	-	-	-
2 (C.A.)	++	++	-	++
3 (C.A. + Ag)	+	-	-	+
4 (Placebo)	++	++	-	++

C.A.: contrast agent; Ag: agmatine. Source: Elaborated by the authors.

## Discussion

The present study delved into the effects of agmatine on contrast-induced nephrotoxicity in rat models. The results demonstrated that agmatine, administered concurrently to contrast exposure, exhibited a protective potential against nephrotoxicity. Our research contributes to the literature investigating agmatine in the context of CIN.

The nephrotoxic effects of contrast agents have been examined in various experimental studies. The underlying mechanism is generally believed to be rooted in vasoconstriction, oxidative stress, and inflammation with critical roles attributed to mediators such as adenosine, nitricoxide, and reactive oxygen species^1,3-5,17,18^. In this current study, elevated levels of inflammatory markers such as TNF-alpha and TGF-beta along with reduced levels of antioxidant enzymes such as GPx, detected in rats administered with contrast agents, may potentially serve as indicators of this condition. The presence of all these factors may explain the cause of renal damage that we have demonstrated biochemically and histopathologically in the group exposed to contrast agents.

Strategies for the prevention or treatment of CIN have become a focal point in contemporary medicine. Despite numerous agents being tested for this purpose, no unequivocally effective preventive method has been identified yet^19-21^.

Considering the etiological factors, we chose to investigate agmatine in our study due to its dual attributes of being both an antioxidant and anti-inflammatory agent, as well as its potential impact on nitricoxide pathways, which we believe could render it effective in CIN. Simultaneous administration of agmatine along side the contrast agent in rats not only reduced cytokine levels but also elevated antioxidant enzyme levels, potentially leading to an amelioration in kidney function and histology.

To the best of our knowledge, there is only another study in the literature that investigates agmatine in the context of contrast nephropathy. In contrary to our current study, Sugiura et al.^14^ did not find agmatine to be effective in CIN in their rat experiments. They attributed this discrepancy to potential challenges in inducing CIN in rodents due to physiological and structural differences of their kidneys. This discrepancy may be primarily attributed to differences in experimental design and model severity.

In the study by Sugiura et al.^14^, a more aggressive CIN model was employed, including unilateral nephrectomy and the pharmacological inhibition of prostaglandin and nitric oxide synthesis using indomethacin and L-NAME. These interventions significantly reduce renal perfusion, potentially overwhelming the protective capacity of agmatine, especially when administered as a single intravenous bolus shortly before contrast exposure. Agmatine was administered via oral gavage at the dose of 20 mg/kg once daily for three consecutive days prior to contrast administration. This oral route was chosen for its translational relevance and its ability to generate more sustained plasma levels compared to bolus parenteral applications.

Pharmacokinetic studies in Sprague Dawley rats have demonstrated that oral administration leads to a prolonged plasma half-life (≈74.4–117 min) and a bioavailability of ~29–35%, compared to much shorter half-lives observed with intravenous injection^22^.

In the study by Sugiura et al.^14^, tubular necrosis was observed following contrast administration, which indicates a more advanced stage of renal injury. In fact, tubular necrosis was also observed in their model, reflecting a higher degree of tissue damage. Typically, as demonstrated in our model, tubular necrosis is not considered a characteristic finding in CIN14. CIN is generally associated with mild, reversible tubular injury rather than overt necrosis. The absence of necrosis in our study may be attributed to differences in the experimental protocol, particularly the lack of additional sensitizing factors such as nephrectomy, prostaglandin inhibition, or nitric oxide synthase blockade, which were present in the prior model. This highlights the importance of experimental conditions in determining the severity of contrast-induced renal damage^14^.

In our study, by contrast, agmatine was administered orally for three consecutive days prior to contrast exposure, allowing for sustained systemic levels and sufficient time for its antioxidant and anti-inflammatory properties to take effect. Additionally, our evaluation was conducted on day 4, capturing both the peak injury and potential early repair processes, whereas the previous study evaluated only at 24 hours, possibly missing delayed protective effects. These findings suggest that agmatine’s renoprotective efficacy is highly dependent on the timing, duration, and context of its administration, and may be more effective in moderate injury models rather than extreme ones.

There are also studies that have investigated the effects of agmatine on other renal diseases^15,16,23,24^. For example, it has been demonstrated that agmatine administered to rats with ischemic acute kidney injury improved renal function and histology^16^. The renoprotective effects of agmatine have been attributed to its activation of imidazoline 1 receptors which suppresses the increased renal sympathetic activity during ischemia^9^. Similarly, in rats with rhabdomyolysis-induced acute kidney injury, as in our study, the use of agmatine was associated with a decrease in blood urea nitrogen, creatinine levels, a decrease in inflammatory cytokines such as TNF-alpha and IL-1 beta and an increase in SOD levels.

Agmatine has also been reported to improve kidney function and histological outcomes in rats with gentamicin induced and cisplatin-induced nephrotoxicity^10,13^. The common consensus from these studies is that the underlying mechanism involves the reduction of oxidative stress by preventing the production of reactive oxygen species, the suppression of inflammatory cytokines, and the regulation of NO levels^15,23,25^. Although not directly related to renal injury, recent evidence suggests that agmatine exerts broad cytoprotective effects through modulation of oxidative stress and inflammatory signaling pathways. A recent study by Tanoğlu et al.^26^ reported significant anti-inflammatory and cellular protective effects of agmatine in colorectal cancer cell models, supporting the notion that agmatine may confer tissue protection through shared molecular mechanisms.

## Conclusion

Our current study has shed light on the potential therapeutic role of agmatine in CIN and under score the significance of continued research into novel strategies for the prevention and treatment of CIN in clinical practice.

The concurrent administration of agmatine along side contrast agents demonstrated promising renoprotective effects, including reductions in cytokine levels, improvements in antioxidant enzyme levels and a likely restoration of kidney function and histology. In contrast to the study by Sugiura et al.^8^, which reported no protective effect of agmatine in CIN, our study demonstrated significant biochemical and histopathological improvements following agmatine treatment. This discrepancy may be primarily attributed to differences in experimental design and model severity. In the study by Sugiura et al.^8^, a more aggressive CIN model was employed, including unilateral nephrectomy and the pharmacological inhibition of prostaglandin and nitric oxide synthesis using indomethacin and L-NAME. These interventions significantly reduce renal perfusion, potentially overwhelming the protective capacity of agmatine, especially when administered as a single intravenous bolus shortly before contrast exposure.

In this current study, by contrast, agmatine was administered orally for three consecutive days prior to contrast exposure, allowing for sustained systemic levels and sufficient time for its antioxidant and anti-inflammatory properties to take effect. Additionally, our evaluation was conducted on day 4, capturing both the peak injury and potential early repair processes, whereas the previous study evaluated only at 24 hours, possibly missing delayed protective effects. These findings suggest that agmatine’s renoprotective efficacy is highly dependent on the timing, duration and context of its administration and may be more effective in moderate rather than extreme injury models.

Our study, together with supporting evidence from other nephrotoxicity and inflammation models, indicates that agmatine holds strong potential as a renoprotective agent in contrast-induced nephropathy.

Its dual antioxidant and anti-inflammatory mechanisms, supported by findings in CIN, methotrexate-induced injury, diabetic nephropathy, and acute pancreatitis models, underscore the significance of continued investigation into its therapeutic role. However, it is essential to acknowledge the scarcity of studies in this field, emphasizing the need for further research, particularly in human subjects.

## Data Availability

The data will be available upon request from the corresponding author.
